# Dual parameter smart sensor for nitrogen and temperature sensing based on defect-engineered 1T-MoS_2_

**DOI:** 10.1038/s41598-024-72632-4

**Published:** 2024-09-14

**Authors:** Mir Sahanur Ali, Mir Sahidul Ali, Subhasish Mallick, Shubhranshu Bhandari, Mir Intaj Ali, Subhenjit Hazra, Bodhishatwa Roy, Sanatan Chattopadhyay, Srikanta Karmakar, Dipankar Chattopadhyay

**Affiliations:** 1 Centre for Research in Nanoscience and Nanotechnology, University of Calcutta, Kolkata, West Bengal 700106 India; 2 Department of Polymer Science and Technology, University of Calcutta, Kolkata, West Bengal 700009 India; 3 The Fritz Haber Research Center, Institute of Chemistry, The Hebrew University of Jerusalem, 9190401 Jerusalem, Israel; 4 Environment and Sustainability Institute (ESI), University of Exeter, Penryn Campus, Cornwall, TR10 9FE UK; 5 Centre for Nanoscience and Nanotechnology, Sathyabama Institute of Science and Technology, Chennai, Tamil Nadu 600119 India; 6 Department of Electronic Science, University of Calcutta, Kolkata, West Bengal 700009 India

**Keywords:** Dichalcogenides, Point defect, Line defect, Gasochromic, Thermochromic, Gas sensor, Materials science, Nanoscience and technology

## Abstract

In general, defects are crucial in designing the different properties of two-dimensional materials. Therefore large variations in the electric and optical characteristics of two-dimensional layered molybdenum disulphide might be attributed to defects. This study presents the design of a temperature and nitrogen sensor based on few-layer molybdenum disulfide sheets (FLMS), which was developed from bulk MoS_2_ (BMS) through an exfoliation approach. The produced sulfur defect, molybdenum defect, line defect, and plane defect were characterized by scanning transmission electron microscopy (STEM), which substantially impacts the sensing characteristics of the resulting FLMS. Our theoretical analysis validates that the sulfur vacancies of the MoS_2_ lattice improve sensing performance by promoting effective charge transfer and surface interactions with target analytes. The FLMS-based sensor showed a high sensitivity for detecting nitrogen gas with a detection limit (LOD) of ~ 0.18 ppm. Additionally, temperature-detecting capabilities were assessed over various temperatures, showing outstanding stability and repeatability. To the best of our knowledge, this material is the first of its kind, demonstrating visible N_2_ gas sensing with chromic behaviour.

**Supplementary Information:**

The online version contains supplementary material available at 10.1038/s41598-024-72632-4.

## Introduction

Among the post-graphene development, due to distinct electrical, optical, and mechanical characteristics, 2D semiconducting transition metal dichalcogenides (TMDCs) are currently on the horizon of materials science and electronics to sensing. Because of its layered structure and semiconducting qualities, molybdenum disulfide (MoS_2_) has become one of the fascinating TMDCs. The exfoliation of MoS_2_ from bulk crystals has emerged as a versatile technique to obtain ultrathin layers of this material, enabling the exploration of its fundamental properties and diverse applications. In this study, we focus on the synthesis of MoS_2_ by the exfoliation method, aiming to elucidate its structural characteristics and exploit specific defects for sensing applications. One of the key findings of our research is the identification and characterization of sulfur, molybdenum, line and plane defects within the exfoliated MoS_2_ layers. These defects, arising from the intrinsic crystal structure of MoS_2_, present unique opportunities for tailoring the properties of material and functionalities. By leveraging these defects, we explore their potential utility in gas sensing, particularly for nitrogen gas detection and temperature sensing applications. The gaseous inertness of nitrogen gas makes it perfect for a variety of technical and manufacturing applications^1,2^. At high partial pressure (4 bar), this non-toxic element N_2_ functions as an anaesthetic agent. In a confined environment, nitrogen can remove oxygen from the surrounding air, posing a risk of asphyxiation. A brief mental impairment caused by nitrogen narcosis, a condition comparable to nitrous oxide intoxication, was reported by Albert Behnke in 1935^3^. Therefore, breathing too much nitrogen gas will result in a variety of physical and mental impairment symptoms. The possibility of undetected leaks in nitrogen-powered systems is a major safety and regulatory problem. Gas sensing is a critical area of research with numerous applications in environmental monitoring, industrial safety, and healthcare. Nitrogen gas, being an inert and abundant component of the atmosphere, poses challenges for traditional sensing approaches. Previously, Laser-induced graphene (LIG) and violet phosphorus have been used for electrochemical detection of plant-available nitrogen (NH^4+^ and NO^3−^) and nitrogen derivative gas^4^. N_2_ gas detection is crucial for laboratory applications and human health issues. Studies on nanomaterial-based detectors have explored advantages and disadvantages, but fabrication can be challenging and expensive. Typically, commercial N_2_ detectors utilize oxygen sensors and use the inverse rule of detected O_2_ gas concentration to estimate the concentration of N_2_ gas. There is plenty of evidence for detecting nitrogen-containing gases or compounds; however, hardly any evidence has been found that demonstrates distinct practical and visible N_2_ gas sensing ability to the best of our knowledge. Visible optical N_2_ sensors are highly effective for leak detection due to their non-electrical connection requirement and are free of spark hazards, making them safe in explosive situations. This reduces the cost of building the sensor and simplifies measurement, especially for inexperienced workers. The development of trouble-free visible-color sensors is vital for the very sensitive, reversible, simple quantification and detection of N_2_, which verifies the safety of end users from such harmful components. Chromogenic materials or “chameleon materials”^5^ are employed regularly in our daily lives^5^. Shengqing et al. reported that nanoporous tungsten oxide (WO_3_) films have gasochoromic capabilities due to their high hydrogen diffusion ability and that WO_3_ has chromogenic qualities when stimulated with H_2_^6^. Also, thermochromic materials applied in the field of visible temperature indicators (thermochromic spoon), sunlight-chromogenic textiles, paints, inks, cosmetics, architecture, smart windows, smart packaging, and optical switches are a few^7,8^.

Overall, this research contributes to the fundamental understanding of few-layer MoS_2_ (FLMS) synthesis and its application in gas and temperature sensing. By harnessing the unique properties of elemental defects, line defects and plane defects, we pave the way for the development of advanced sensing technologies with enhanced performance and functionality. The inert nitrogen gas sensing performance was evaluated at room temperature (RT), revealing a quick response and recovery with great sensitivity down to 0.18 ppm. Finally, using density functional theory (DFT), an exhaustive sensing mechanism is proposed based on the favourable adsorption sites present at the edges and in-plane sites of FLMS and their interaction energy. Our built sensor is not only able to sense nitrogen gas but also detect temperature over a range through an Android application. The FLMS shows a chromic effect when it is exposed to N_2_ gas or heat; using this property, we have built a visible dual-parameter visible sensor. This visible sensor is spark-free and can be handled by inefficient fellows, which is the main advantage of this sensor.

## Materials and methods

**Fig. 1:**
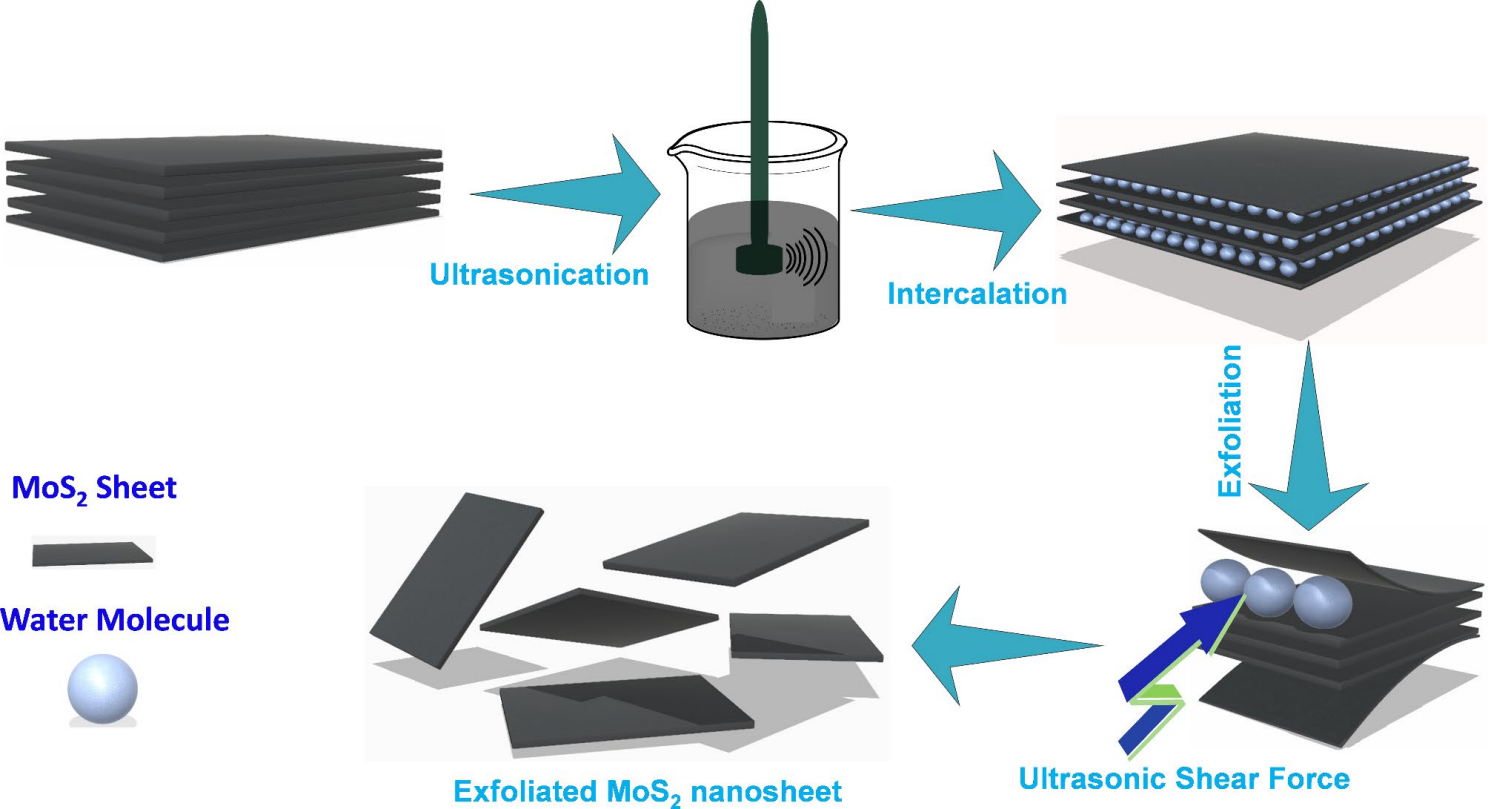
Schematic of synthesis procedure of a few layers of MoS_2_ by ultrasonic wave exfoliation.

Sisco Research Laboratories (SRL) supplied Molybdenum Disulfide (MoS_2_, 99.999%), and our laboratory produced triple distilled water (TDW). Without additional purification, all of the reagents were employed. The bulk MoS_2_ (BMS) powder was subjected to a straightforward probe sonication in TDW to synthesize the few-layer MoS_2_ nanosheets. In a typical synthesis procedure, 200 ml of TDW was used to dissolve 500 mg of commercial BMS powder, and the mixture was then agitated for 30 min to create a homogenous solution. The homogeneous BMS solution was then ultrasonically processed for 16 h before being left undisturbed for the night. A light greenish-colored solution was then isolated from the upper portion of the centrifuged solution after the upper portion of the solution had been centrifuged for 10 min at 5000 rpm. The synthesis mechanism is represented in detail in Fig. 1.

### Mechanism of formation of few-layer MoS_2_

By using weak van der Waals (vdW) forces, BMS is produced by stacking monolayer MoS_2_ with a thickness of around 0.65 nm. Solvents have an important role in the exfoliation of BMS due to factors such as dispersion behaviour and solvation energy. Using the mixing enthalpy theory, it can be explained. In the beginning, the water molecule in the BMS was confined between two layers of 2D MoS_2_ sheet. However, the surface energy of water is lower than that of a BMS flake; therefore, hydrophobic force and vdW interactions between two neighbouring sheets tend to exclude the trapped water molecules to minimize the system’s free energy. This interlayer vdW interaction and hydrophobic force have been overcome through the introduction of ultrasonic waves. In this situation, the confined water molecule starts to migrate into two neighbouring sheets^9,10^. The expansion of interlayer through intercalation has also been reported by Chen et al.^11^. It is well known that defect formation energy for displacing the chalcogen atom is significantly higher than for displacing transition metal; therefore, ultrasonic wave energy is thought to be responsible for the formation of sulfur vacancy (SV) in exfoliated MoS_2_ nanosheet.

### Fabrication of gas sensor and measurement technique

**Fig. 2:**
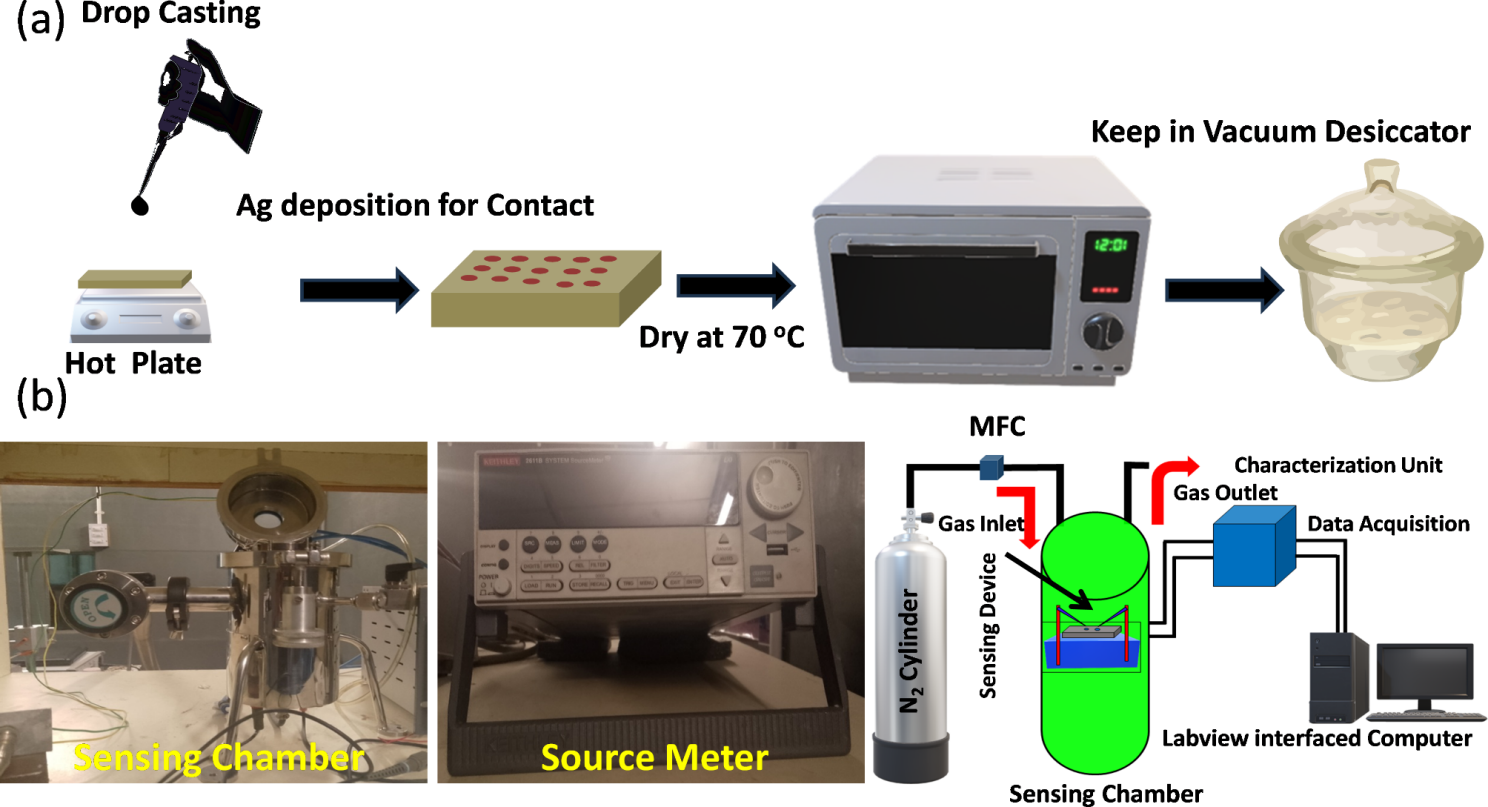
(**a**) Schematics of the process of fabrication of FLMS-based films; (**b**) Schematic of the experimental setup for N_2_ gas sensing

The gas sensor was fabricated using a thin film, and the relevant fabrication method of the film is shown in Fig. 2a. When the exfoliation process was completed, the final solution was coated on a square glass substrate (2 cm × 2 cm) by drop casting method. Subsequently, to fabricate the resistive sensor, two Ag electrodes were deposited onto the deposited film using the thermal evaporation method. After that, the coated sensor sheet was placed into a glass dish and placed in an oven at 35 °C for 5 h. The sensor was mounted inside a closed chamber, and resistance was recorded using a source meter (Keithley 2611B) connected to a computer. The concentration of N_2_ was varied from 20 to 160 ppm using a mass flow controller (MFC) and the entire experiment was performed at room temperature (300 K). Between N_2_ pulses, the gas chamber was purged with air, allowing sensor surface recovery to atmospheric conditions. The schematic of the experimental setup is represented in Fig. 2b.

## Results and discussion

**Fig. 3:**
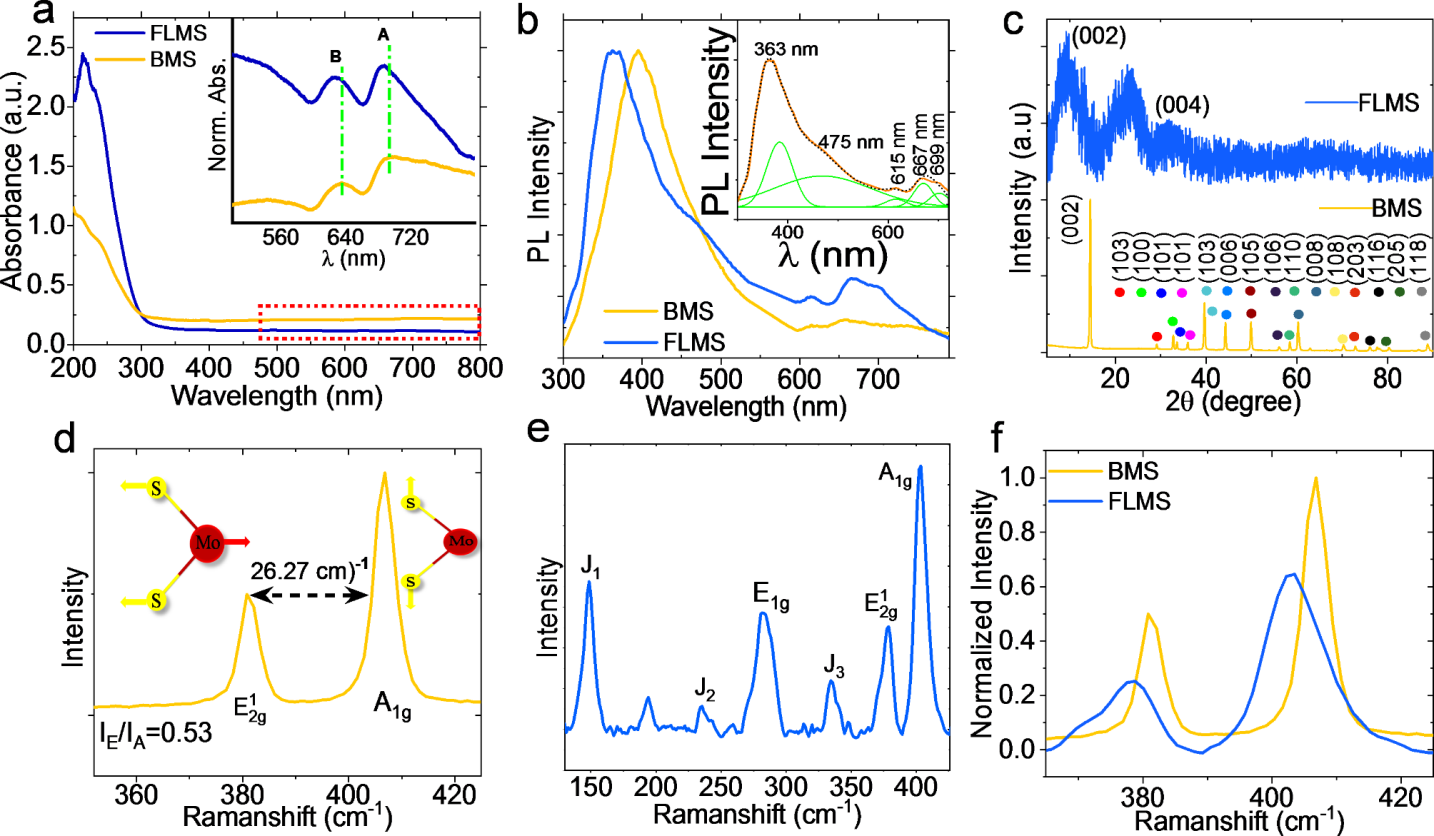
(**a**) UV-Vis spectra of BMS and FLMS (**b**) PL emission spectra of BMS and FLMS-based thin film; its inset showed the Gaussian fitted PL emission spectrum of FLMS-based film. (**c**) XRD spectrum of BMS and FLMS film, (**d**) Raman spectra of BMS, (**e**) Raman spectra of FLMS-based thin films, (**f**) FWHM and intensity comparison of E_2g_ and A_1g_ peak of BMS and FLMS.

In Fig. 3a, the UV-Vis spectra of bulk MoS_2_ (BMS) reveal two excitonic peaks, “A and B”, at 630 nm and 690 nm and that of few-layer MoS_2_ (FLMS) at 625 nm and 685 nm, respectively. The excitonic peaks “A and B” are caused primarily by the transition between the split-valence band and the conduction band at the K-point of Brillouin zone^12,13^. An electron from the bottom of the conduction band (CB) and a hole from the top of the valence band (VB) interact to generate the A exciton via Coulomb interaction. The Coulomb interaction between an electron from the bottom of the CB and a hole from the split VB lower level produces the B exciton^12,13^. In the case of BMS, the energy separation between these two peaks is mostly due to the spin-orbit interactions and interlayer coupling, whereas in the case of FLMS, the interlayer coupling is reduced^12^. Slight blue shiftings of the two excitonic peaks are observed in the case of FLMS in comparison to BMS (see inset of Fig. 3a). Figure 3b shows the photoluminescence (PL) emission spectra (λ_ex_ = 280 nm) of the BMS and FLMS thin films, which exhibit numerous excitonic peaks, including A, B, C, and D. The inset of Fig. 3b displays the PL emission spectrum of the FLMS-based thin film after Gaussian fitting. PL spectra of FLMS are substantially identical to monolayer MoS_2_ in nature, having excitonic peaks A, B, C, and D at 667 nm, 615 nm, 475 nm, and 363 nm, respectively. These peaks resulted from the Brillouin zone’s transition between several K points^14^. A is produced from a straight band gap transition, whereas B is derived from valence band splitting driven by strong spin-obit coupling^12,14^.

Figure 3c shows the XRD pattern of BMS, which indicates a strong peak matching the (*002*) plane and other weak peaks that are precisely matched with rhombohedral MoS_2_ (JCPDS No: 06–0097). The 2θ = 14.2° (interspace 0.63 nm) corresponds to (*002*) and is widely known as an indicative peak of pure 2 H-MoS_2_^15^. Figure 3c showed broadened signals at 2θ = 9.3° (interlayer spacing ~ 9.0 Å) and 2θ = 32.2°, assigned to (*002*) and (*004*) planes of the MoS_2_ 1T phase^16^. The intercalation of the H_2_O molecule increases the inter-layer spacing of the neighbouring layers, which is responsible for the indicative peak shifting. The presence of stacking defects^17,18^ among MoS_2_ layers was indicated by the asymmetric nature of the reflection at 2 = 32.2°, which could be attributable to H_2_O intercalation. The entry of water molecules into the S-Mo-S layer of 2 H-MoS_2_ may trigger the creation of the 1T MoS_2_ phase. As reported in the prior literature, the 2 H-MoS_2_ interlayer distance increasing from 6.3 to 9.8 Å is clearly evidence of the intercalation process^15–17,19^. The enlarged signal at the plane (*002*) and disappearance of several peaks are owing to the absence of constructive interference from the crystal planes, indicating that BMS peeled to a few layers while detecting signal at the plane (*002*) suggests that exfoliation has no effect on crystallinity^17,20^. According to Gao et al., the intercalation of oxidized DMF species into two S-Mo-S layers causes the interlayer gap to increase^17^. According to another article, water molecules, Li ions, and ammonium ions (NH_3_/NH_4_^+^) intercalated to create 1T MoS_2_^15^.

The 1T phase of MoS_2_ produced from 2 H-bulk MoS_2_ was further identified using Raman spectroscopy. The Raman spectra of BMS (Fig. 3d) show two lattice vibration peaks at 380 cm^− 1^ and 407 cm^− 1^ attributable to in-plane (E_2g_^1^) and out-of-plane (A_1g_) vibrations of the S and Mo atoms, respectively^21–23^. The in-plane Mo-S phonon mode (E_2g_^1^) and the out-of-plane Mo-S mode (A_1g_) are seen at 378.5 cm^− 1^ and 402.8 cm^− 1^, respectively, in the Raman spectra of FLMS (Fig. 3e). The strong octahedral coordination of Mo in 1T MoS_2_ is confirmed by the E_1g_ band at 284 cm^− 1^ and the relatively faint E_2g_^1^ band^24^. Longitudinal acoustic phonon modes of 1T phase MoS_2_ were observed at 334.8 cm^− 1^ (J_3_), 235.05 cm^− 1^ (J_2_), and 148.2 cm^− 1^ (J_1_), which were likewise linked to the creation of 1T MoS_2_ super-lattice structure^24–26^. The presence of J_2_ Raman mode indicates the formation of defect in FLMS^27^. The number of layers in MoS_2_ is determined by the energy difference between the two peaks (E_2g_^1^ and A_1g_). The peak difference of E_2g_^1^ and A_1g_ for BMS is 26.27 cm^− 1^, corresponding to more than 20 layers, whereas, for FLMS, this energy difference is 24.01 cm^− 1^, indicating that FLMS has few layers MoS_2_. Figure 3f shows that the intensity of the BMS Raman peak is significantly higher than that of the 1T MoS_2_ peak. In the case of 1T MoS_2_, the FWHM of the A_1g_ peak becomes wider than in the case of 2 H-MoS_2_. Due to the existence of sulfur vacancy (SV) in 1T MoS_2_, the A_1g_ peak is more pronounced and has a lower intensity when compared to 2 H-MoS_2_^15,26,28^. As a result, Raman spectroscopy explores whether the intercalation of water molecules and the extended sonication period are adequate to produce 1T phase as well as SV, which is discussed later.

**Fig. 4:**
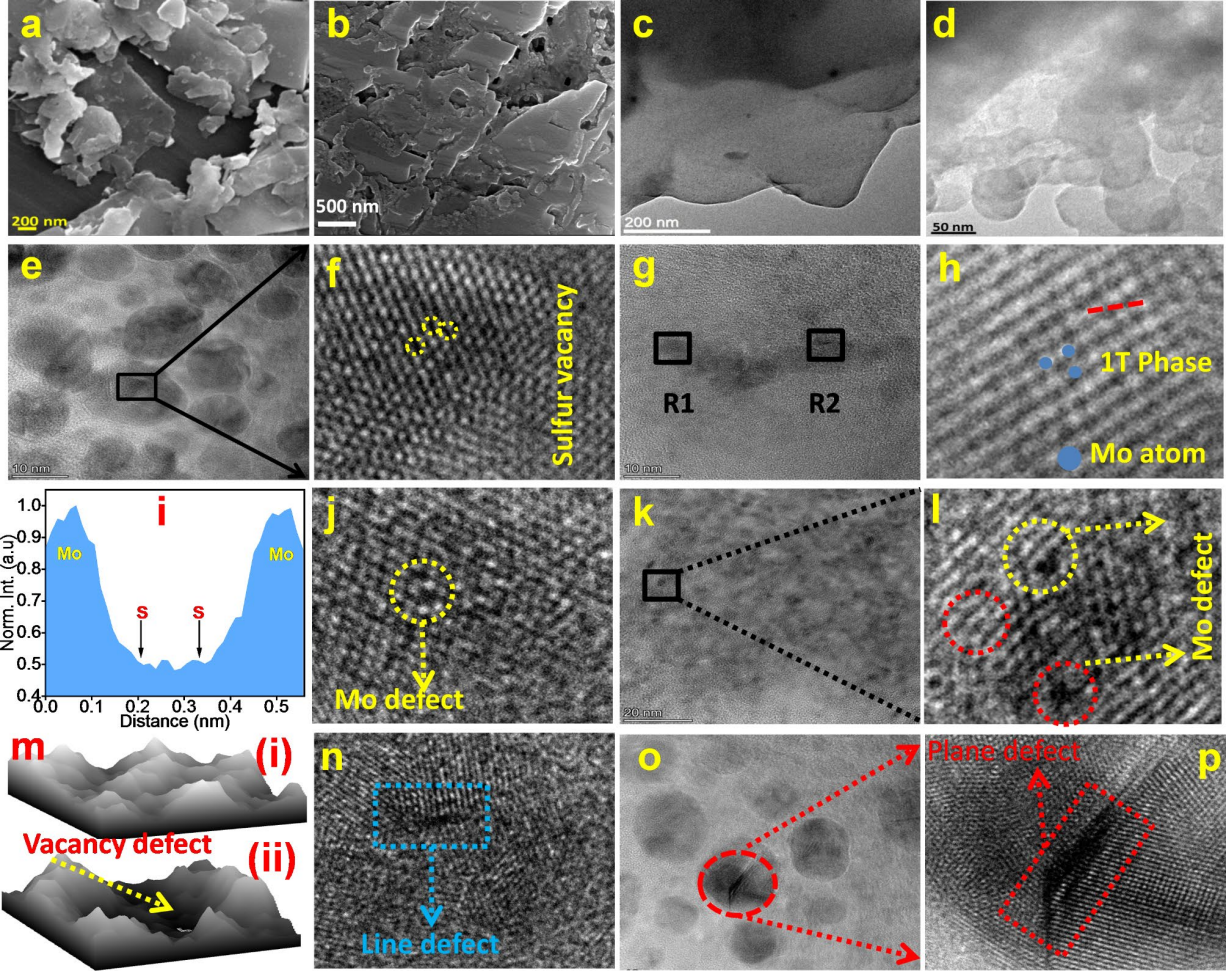
FESEM image of (**a**) BMS (**b**) FLMS, (**c**)-(**d**) TEM image of BMS and FLMS, respectively, (**e**) HRTEM image of the selected portion of FLMS, (**f**) FFTplot of marked region of figure **e**, shows V_s_, (**g**) HRTEM image of different region of MoS_2_ nanosheet containing both V_Mo_ and line defect, (**h**) FFT plot of figure **g**, shows 1T phase of MoS_2_ (blue sphere indicates Mo atoms), (**i**) atomistic line profile of showing modulation of sulfurcontrast, FFT image of marked region of figure (**g**) represent (**j**) V_Mo_, (**k**) line defect, (**l**) HRTEM image of different region of MoS_2_ nanosheet containing V_Mo,_ (**m**) surface plot of R1 of figure (**g**) represent (i) without defect region, (ii) V_Mo_ region, (**n**) FFT image of Fig. 3g (R2) containing line defect, (**o**) HRTEM image of different region of MoS_2_ nanosheet containing two dimensional defect, (**p**) FFT image of red circled region of figure o which clearly shows two dimensional defect.

FESEM, TEM and scanning transmission electron microscopy (STEM) were used to analyze the morphology, structure and defect of the crystals of BMS and FLMS films. The FESEM image of FLMS (Fig. 4a,b) reveals a very thin MoS_2_ flakes sheet with conventional lateral dimensions, while an irregular morphology with a high density of sprayed MoS_2_ flakes with no gaps between them is observed in FESEM image of BMS (Fig. 4a). Transmission electron microscopy (TEM) images of the bulk and FLMS flakes, depicted in Fig. 4c,d respectively, reveal a sheet-like structure. It is clear from the contrast of both TEM images that the thickness of BMS is much higher than that of FLMS. This implies that effective exfoliation of BMS produces a thin sheet, which is consistent with Raman and XRD investigations. STEM was utilized to examine the structural features and flaws in the FLMS. There were characteristics in the STEM-HAADF picture that could be identified as being associated with several kinds of atomic-scale defects in the MoS_2_ lattice, such as molybdenum vacancies, sulfur vacancies, and line and plane defects. Figure 4e represents the STEM image of FLMS, in which sulfur vacancies were observed. The FFT images of Fig. 4e,f represent the sulfur vacancies as dark patches inside the MoS_2_ lattice and as localized intensity variation (yellow circle) in the STEM-HAADF picture. Interestingly, in Figs. 1T and 4g phase of MoS_2_ and Mo defect was detected in a single sheet, which is clearly shown in the FFT images of R1 and R2 in Fig. 4g. Figure 4h reveals the 1T phase of MoS_2_, the corresponding intensity of sulfur and molybdenum atom along the red line plotted in Fig. 4i, which shows that the calculated intensity ratio between 2 S and Mo site is less than 0.25. The Mo vacancy is predicted in Fig. 4j (FFT image of Fig. 4g (R1)) and Fig. 4l (FFT image of Fig. 4k) through the STEM’s Z-contrast mechanism. The surface plot of Fig. 4i is depicted in Fig. 4m, in which Mo defect can be seen clearly. In addition to point defects also, line defects and plane defects were observed in synthesized FLMS. The line defect is presented in Fig. 4n (FFT image of Fig. 4g (R2)), which appeared as distinct contrast variations. The plane defect was characterized by abrupt changes in the intensity contrast and atomic arrangement which is represented in Fig. 4o,p (FFT image of red circled region of Fig. 4o).

### Gas sensing performance

**Fig. 5:**
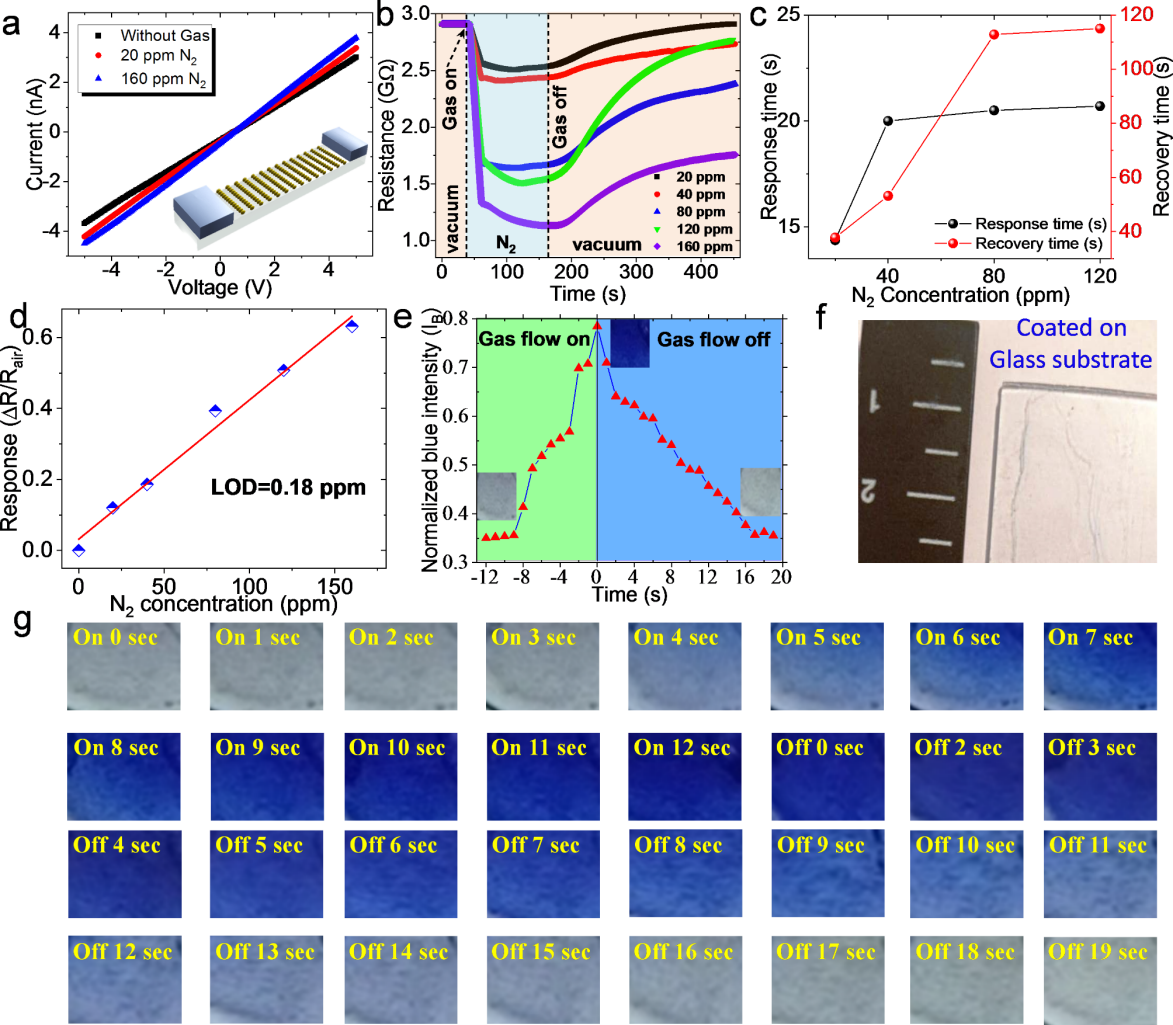
(**a**) I − V dependence of the Ag − MoS_2_ − Ag device at various conditions, (**b**) Sensing R-t plot showing the N_2_ gas ‘on’ and gas ‘off’ condition during the N_2_ sensing experiment at RT, (**c**) Response and recovery time profile at RT, (**d**) Dependence of the amplitude of resistance change ΔR/R_N2_ on the concentration of N_2_ gas, (**e**) The normalized blue intensity vs. gas flow time profile, (**f**) digital image of FLMS, (**g**) digital image of FLMS at different instant of time during N_2_ gas flow.

Figure 5a depicts a schematic of a MoS_2_ channel with Ag electrodes, referred to as an Ag-MoS_2_-Ag device. In the beginning, the current-voltage (I-V) characteristic is measured to examine the electrical and physical connection of the channel. Figure 5a displays the I–V curves of the fabricated FLMS-based film. The measurements were conducted at room temperature (300 K), and bias across the device was set to 2 V for all of our measurements. Figure 5a confirms that the current almost linearly increased with increasing voltage from − 5 to 5 V, which proves that there is an Ohmic contact between the MoS_2_ with metal-electrodes having a resistance of giga order (10^9^). It was also observed from the I-V curve that the current flow is higher in the case of the N_2_ environment than the normal vacuum environment, i.e. increasing in conductance, which is consistent with p-type materials. Figure 5b represents the variation of measured resistance at different conditions of gas flow. The first highlighted regions show the resistance value at vacuum, which can be accepted as the steady state base resistance. We conducted N_2_ gas sensing measurements for the five different N_2_ concentrations (20, 40, 80, 120, and 160 ppm) at room temperature (RT) to determine the best performance of the sensor. The sensor resistance significantly lowers after N_2_ exposure.

Upon exposure to N_2_, the resistance of the sensor decreases significantly because of the electron acceptor nature of N_2_ due to the unpaired electron on the nitrogen atom. The sensing mechanism relies on the direct charge transfer between N_2_ and FLMS. Nitrogen gas is a known electron acceptor due to the unpaired electron on the nitrogen atom. Upon N_2_ adsorption, since the electron extraction from FLMS is causing a decrease in sensor resistance, the FLMS exhibits a p-type characteristic. During subsequent exposure to clean air, the sensor resistance quickly recovers as N_2_ molecules desorb from the surface. This behavior is consistent with the charge transfer mechanism of p-type MoS_2_ gas sensors^21,29^. However, understanding the complete mechanism is a complex subject in gas sensing studies because of the effects of physisorption and the role of defect sites. The FLMS sensors, the adsorption of gas, can be regarded as high energy binding sites (vacancy defects) since they show quick rates of response and recovery at room temperature, consistent with our DFT calculation. There are a number of causes behind the p-type nature of FLMS, including its 1T phase and synthesis-related defects^21,30^. Without any external stimulation, it has been seen that the sensor fully recovers up to 120 ppm N_2_ gas concentration at room temperature. The response and recovery profile (Fig. 5c) shows that response time initially increases with increasing N_2_ concentration and then nearly equalizes for 40–120 ppm N_2_ concentration. The response nature differs slightly from the nature of recovery; the recovery time rises with N_2_ concentration and nearly remains constant for 80 and 160 ppm. The quick response and recovery times at RT, as shown in Fig. 5c, point to defect-dominated physisorption. The equation below can be used to get the sensitivity (S) at RT^29,31^.

1 $$S=\frac{{\left( {{R_{gas}} - {R_{air}}} \right)}}{{{R_{air}}}}$$

Figure 5d depicts the functional relationship between the concentration of N_2_ and the gas-sensing response of 1T-MoS_2_. The results demonstrate a significant linear relationship between responsiveness and various concentrations. The detection limit (DL) and selectivity of the sensors are critical components that must be explored for application in real life. Importantly, based on the linear fitting, the limit of detection (DL) of the device can be estimated as^32^

2 $$DL=\frac{{{\text{3}}\sigma }}{{\text{s}}}.$$

Where ‘*s*’ is the slope of the linear fit and σ is the standard deviation, giving our device a limit of detection (LOD) to N_2_ of ~ 0.18ppm, which is rather small and suitable for practical use.

The N_2_ detector was fabricated by simply coating the FLMS with an evaporation method on a standard glass substrate. When N_2_ gas is introduced to the FLMS film, a gasochromic signal that can be seen with the naked eye is identified. Notably, it is discovered that gasochromic detection is limited to a solid phase and not a liquid phase. Within 1 s of being exposed to N_2_ gas, a fast color shift occurred. We employ the RGB color triplet approach to examine the responsiveness and recovery of FLMS film with N_2_ gas. Images were taken with a smartphone camera with manual focus in all cases. Matlab software was used to extract the RGB values, which indicate the intensity of the red, green, and blue hues, from each image. The variables r, g, and b of the EXG index are normalized values of the red, green, and blue channels, respectively, according to Equation. ^33^.

3 $$r=\frac{{{R_N}}}{{{R_N}+{G_N}+{B_N}}}$$

4 $$g=\frac{{{G_N}}}{{{R_N}+{G_N}+{B_N}}}$$

5 $$b=\frac{{{B_N}}}{{{R_N}+{G_N}+{B_N}}}$$

6 $${R_N}=\frac{R}{{{R_{\hbox{max} }}}},{G_N}=\frac{G}{{{G_{\hbox{max} }}}},{B_N}=\frac{B}{{{B_{\hbox{max} }}}}$$

Here, the normalized values of each band are *R*_*N*_, *G*_*N*_, and *B*_*N*,_ and the non-normalized values are R, G, and B of the red, green, and blue channels, respectively; and *R*_*max*_= *G*_*max*_ = *B*_*max*_ are the maximum digital numbers for each channel (255 on the 0–255 scale).

To establish the gasochromic reaction and recovery of FLMS, we set up a plot of film between variables *b* and gas flow time (Fig. 5e). We have noted that when FLMS film is exposed to N_2_ gas, the FLMS film shows intense blue color intensity. Sefaattin et al. showed that in the case of monolayer MoS_2_, luminescence is due to physisorbed N_2_ molecules^34^. Figure 5e shows that a chromic signal may be seen with bare eyes in 4 s and reaches a saturated value of b in 12 s. We have also seen that the chromic effect is reversible at room temperature in the absence of any stimulus like optical or thermal sources; this implies that the contact between FLMS and N_2_ molecules is weak vdW, i.e. physisorbed at the defect location^34,35^. The measured recovery time is 19 s, which is significantly longer than the response time and is consistent with the electrical measurement discussed subsequently. Unskilled personnel can readily handle the FLMS film sensor due to its quick response and recovery time. The digital picture of FLMS-based film and FLMS film under N_2_ exposure are shown in Fig. 5f and g, respectively.

**Fig. 6:**
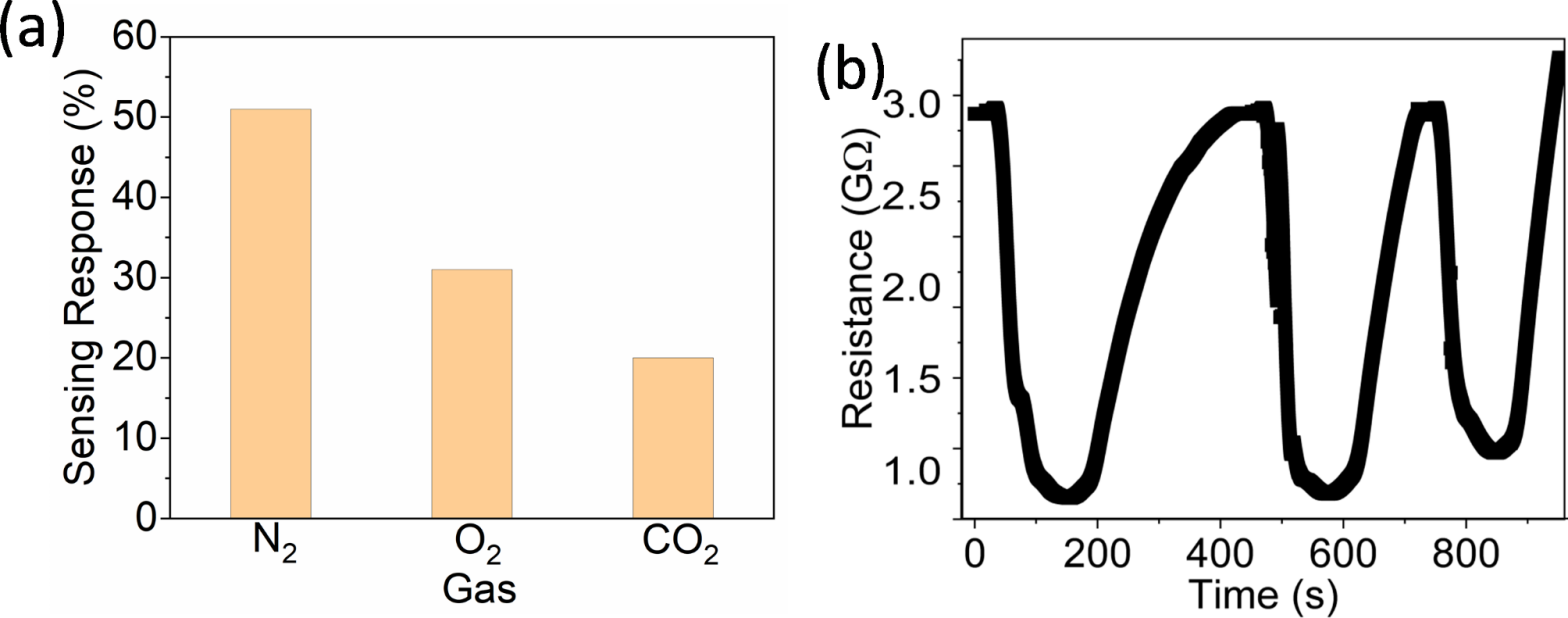
(**a**) Histogram plot showing the room temperature response of the FLMS-based sensor for N_2_, O_2_ and CO_2_, (**b**) Long-term stability of FLMS-based gas sensor to 120 ppm N_2_ at RT.

In order to determine the selectivity of the FLMS-based film for the N_2_ sensing performance, sensors were exposed to carbon dioxide (CO_2_) and oxygen (O_2_). Figure S1 (in ESI) displays the sensing response curves of other gases at bias voltage 2 V and RT. This demonstrates how exposure to gases can affect the behavior of FLMS-based film. A decrease in conductance was observed in the case of O_2_ and CO_2_ gases, while an increase in conductance was observed in the case of N_2_ gas. As seen in Fig. 6a, the sensor has less CO_2_ and O_2_ sensing response than N_2_. This is likely the reason why the FLMS-based film has good selectivity for N_2_ sensing against major abundant naturally occurring gas CO_2_ and O_2_. Repetitive performance evaluation is essential for assessing the stability and long-term dependability of gas sensors. Using several cycles of gas exposure and recovery, we used a standardized testing strategy to evaluate the repeating performance of the gas sensor. To demonstrate the significance of the FLMS-based reversibility of the gas sensor, a cyclic test up to 150 ppm N_2_ is conducted at RT. As seen in Fig. 6b, the response to N_2_ exhibits a modest attenuation of ≈ 2% across 3 cycles, suggesting that p-type MoS_2_ stability has to be enhanced. MoS_2_ partial surface oxidation is most likely the source of this performance loss.

### Luminescent thermo-sensor

**Fig. 7:**
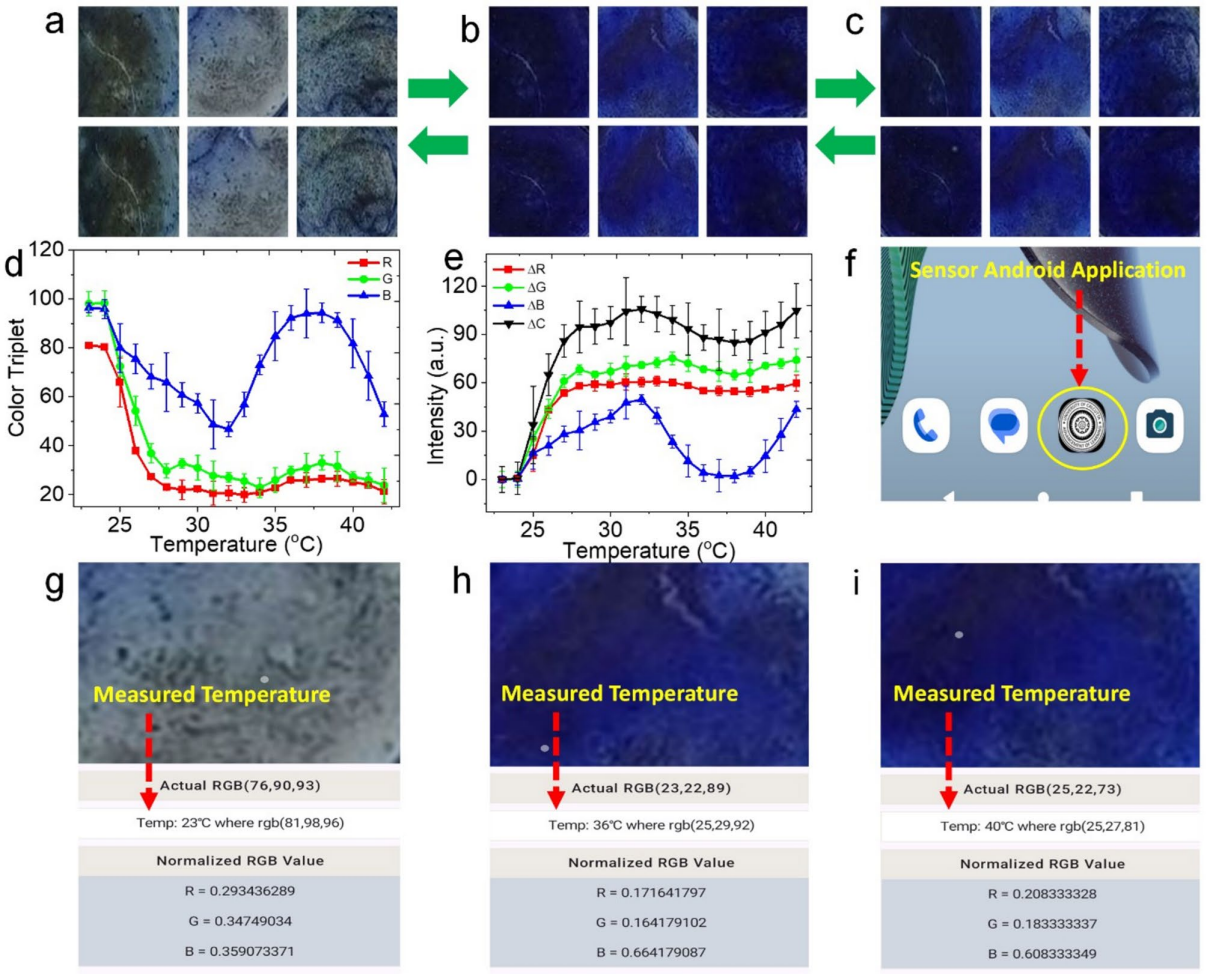
Digital images of the FLMS-based thin film at different temperatures in the open atmosphere (a) 23 °C, (b) 36 °C, and (**c**) 40 °C; (**d**) color triplet of the separate *R*, *G*, and *B* values at different temperatures with errors; (e) Response of the separate *ΔR*, *ΔG*, and *ΔB* values at different temperatures with errors, (**f**) screenshot image of icon of installed android application in mobile phone; screenshot image of sensing performance of android application at (**g**) 23 °C, (**h**) 36 °C, and (**i**) 40 °C.

The colour of the digital photographs of the BMS and FLMS-based thin films was taken at different temperatures ranging from RT (23 °C) to 42 °C using a smartphone fixed at a distance of 20 cm. Three sets of the thin film were placed above a hot plate, and the corresponding temperature was recorded using a digital thermometer. The thermochromic color change was observed by the naked eye, as shown in Fig. 7a–c. Both increasing and lowering temperatures were used to take the digital picturescolor analysis was performed with Adobe Photoshop and Matlab software, using RGB values with an 8-bit resolution (256-bit color space, where white is represented by 255, 255, 255 and black by 0 0 0)^36^. The Euclidean distance equation is commonly used to represent data as total color differences (C)^36,37^.

7 $$\nabla C=\sqrt {{{\left( {\nabla R} \right)}^2}+{{\left( {\nabla G} \right)}^2}+{{\left( {\nabla B} \right)}^2}}.$$

Here, *ΔR*, *ΔG*, and *ΔB* are the changes in *R*, *G*, and *B* colors from reference values, respectively. The digital photos of the FLMS thin films obtained at various temperatures showed a distinct color change, but the BMS-based thin film did not show any change. As the temperature was raised from room temperature (RT; 23 °C) to 35 °C, there was a noticeable change from transparent film to a deep bluish color. The color of blue was somewhat reduced after 35 °C, and no further increase in temperature was detected. The RGB values were then taken from the Matlab software, and the relative changes were shown in Fig. 7d and e to determine this color change in terms of color triplet. Initially, as temperature increased, the RGB values declined, and rapid shifts occurred at 32 °C. Following that, the values of B reached at a considerably faster rate than the values of RG. At 35 °C, there was a distinct peak. Further, *ΔR*, *ΔG*, *ΔB*, and *Δc* values (Eq. 7) were calculated and variations are shown in Fig. 7e. This finding suggests that the FLMS film was extremely temperature sensitive in the low-temperature zone.

A mobile smartphone running the Android operating system was used to take pictures using its built-in camera, ensuring consistent lighting and framing for precise color temperature measurement. The Android Studio software was developed with Android OS through Kotlin language. After obtaining the pictures of the FLMS-based film, the image processing is divided into many stages. When the user wants to measure the unknown temperature of an FLMS-based film, the user may capture a photograph of the film, load it from the gallery, and mark the colored area on the photograph. The app then uses the trained model along with a color-matching algorithm to calculate the temperature level of the FLMS-based film. The colorimetric tests in Fig. 7a, b, and c were first processed from the collected photos and stored with their associated temperature values in order to test the Android app and the color-matching algorithms. The image was uploaded in the app from the gallery and showed the temperature of the film, which was consistent with the real temperature. The screenshot of the app is shown in Fig. 7f–i, which is showing the corresponding temperatures.

## Theoretical results and discussions

**Fig. 8:**
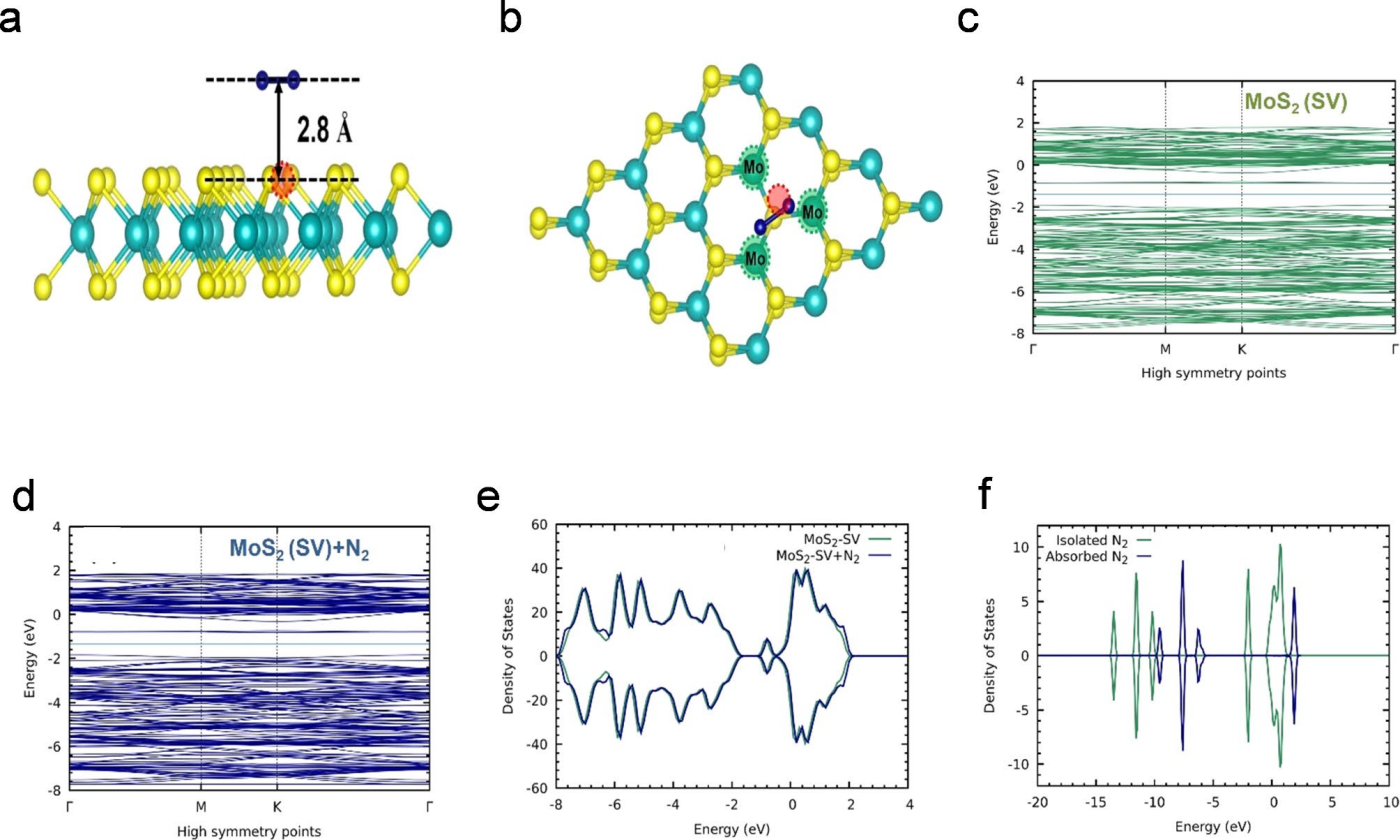
The structure of the N_2_ adsorbed defected MoS_2_ (SV) monolayer from (**a**) side view and (**b**) top view. The cyan and yellow balls represent Mo and S atoms, respectively. The red dotted circle denotes the sulfur vacancy site. The unsaturated Mo atoms near to SV are denoted as a green dotted circle. Band structures for the (**c**) MoS_2_ (SV) monolayer and (**d**) N_2_ adsorbed MoS_2_ (SV) monolayer. The cyan line indicates the Fermi energy level of TDOS and PDOS for (**e**) the N_2_ adsorbed MoS_2_ (SV) (in blue color) compared with MoS_2_ (SV) (in green color), and (**f**) isolated N_2_ (in green color) compared with the adsorbed N_2_ (in blue color).

To gain insights into the adsorption behaviour of N_2_ on the MoS_2_ (SV) monolayer, we initially placed the N_2_ at varying distances and orientations from the SV site and optimized their configurations to obtain the most stable geometry. The calculations indicate that N_2_ can be physisorbed on MoS_2_ (SV) at a distinct height from the SV site, and the presence of van der Waals interaction is detailed in supporting information. Figure 8a,b visually illustrate the optimized geometry that represents the most stable configuration for adsorbed N_2_. The optimized geometry shows that unsaturated Mo atoms surrounding the single SV relaxed a little towards the SV site. The Mo-S bond lengths near SV site reduced to 2.39 Å from 2.42 Å in a perfect MoS_2_ monolayer (see Fig. S2 in ESI), which is aligned with prior research^38,39^. Figure 8c,d illustrate that the presence of SV in MoS_2_ induces defect states within the band gap region, primarily originating from the three unsaturated Mo atoms located near the SV site. Consequently, the calculated band gap of MoS_2_ (SV) is notably reduced to 1.04 eV, a significant narrowing compared to the pristine MoS_2_ monolayer, which has a band gap of 1.65 eV when using the same pseudo potentials, as shown in the Electronic Supporting Information (ESI). For a more comprehensive analysis, we have generated the total density of states (TDOS) for the N_2_-adsorbed MoS_2_ (SV) monolayer and compared it with the TDOS of the MoS_2_ (SV) monolayer in Fig. 8e. Additionally, we have examined the TDOS for isolated N_2_ and the partial density of states (PDOS) for adsorbed N_2_ in Fig. 8f. In Fig. 8e, noticeable changes are observed after N_2_ adsorption, particularly around − 6.2 eV and − 7.9 eV in the TDOS, corresponding to the valence band. Moreover, shifts towards higher energy levels are observed in the conduction band, primarily attributed to the electron density associated with N_2_. Figure 8f reveals that upon adsorption, the occupied states of N_2_ shift to higher energy levels. Interestingly, in the unoccupied states, a single peak emerges, contrasting with the distinct peaks observed in isolated N_2_. The adsorption energy (*E*_*ads*_) for N_2_ is calculated to be −3.61 kcal/mol, surpassing the *E*_*ads*_ for N_2_ on the pristine MoS_2_ monolayer (-2.54 kcal/mol), signifying that the presence of SV enhances the adsorption strength of MoS_2_. Charge transfer analysis demonstrates that the N_2_ molecule gains 0.01 e charges from MoS_2_ (SV), indicating the role of N_2_ as a charge acceptor, while MoS_2_ (SV) acts as a charge donor.

## Conclusion

In summary, this work reveals the thermochromic and gasochromic nature of defect-engineered 1T MoS_2_ and explores its applicability as a temperature sensor as well as a nitrogen sensor, i.e., a dual sensing platform. Our prepared FLMS-based sensor shows rapid response to N_2_ with LOD ~ 0.18 ppm; also, this sensor exhibits rapid recovery up to 120 ppm. Overall, this study shows that 1T MoS_2_ with defects has exciting opportunities for the development of effective, versatile sensors for environmental monitoring, space science and other applications.

## Electronic supplementary material

Below is the link to the electronic supplementary material.

Supplementary Material 1

## Data Availability

The original data which supports the findings of current study are available from the corresponding author upon request.
