# Supplementary material for: Dual parameter smart sensor for nitrogen and temperature sensing based on defect-engineered 1T-MoS2
Source: Sci Rep. 2024 Sep 14;14:21469. doi: 10.1038/s41598-024-72632-4 (PMC11401939; doi:10.1038/s41598-024-72632-4)
Supplement: Supplementary file 1 — Supplementary Material 1 [file 41598_2024_72632_MOESM1_ESM.docx]

**Dual Parameter Smart Sensor for Nitrogen and Temperature Sensing Based on Defect-Engineered 1T-MoS_2_**

Mir Sahanur Ali^1,2^, Mir Sahidul Ali^2^, Subhasish Mallick^3^, Shubhranshu Bhandari^4*^, Mir Intaj Ali^5^, Subhenjit Hazra^5^, Bodhishatwa Roy^6^, Sanatan Chattopadhyay^1,6^, Srikanta Karmakar^2*^, Dipankar Chattopadhyay^1,2*^

^1^Centre for Research in Nanoscience and Nanotechnology, University of Calcutta, Kolkata, 700106, West Bengal, India

^2^Department of Polymer Science and Technology, University of Calcutta, Kolkata, 700009, West Bengal, India

^3^The Fritz Haber Research Center, Institute of Chemistry, The Hebrew University of Jerusalem, Jerusalem 9190401, Israel.

^4^Environment and Sustainability Institute (ESI), Penryn Campus, University of Exeter, Cornwall, TR10 9FE, UK

^5^Centre for Nanoscience and Nanotechnology, Sathyabama Institute of Science and Technology, Chennai, Tamil Nadu, 600119, India

^6^Department of Electronic Science, University of Calcutta, Kolkata, 700009, West Bengal, Indi

* Corresponding authors: Tel: +91-9433379034

E-mail: [dipankar.chattopadhyay@gmail.com](mailto:dipankar.chattopadhyay@gmail.com), dcpst@caluniv.ac.in

[shubhranshu0094@gmail.com](mailto:shubhranshu0094@gmail.com), [karmakarsrikanta71@gmail.com](mailto:karmakarsrikanta71@gmail.com)

**Materials Characterizations**:

A UV-Visible spectrometer (Perkin Elmer, lambda25) was used to measure the transmittance of the as-prepared thin film at wavelengths ranging from 200 nm to 700 nm. A photoluminescence spectrofluorimeter (HoribaFluorolog 3-22 luminescence spectrometer) was used to measure the photoluminescence emission of BMS and thin film. TEM images of the synthesized samples were captured using a transmission electron microscope (JEOL JEM 2100 HR, 200kV).The scanning transmission electron microscopy (STEM), high resolution transmission electron microscopy(HRTEM) and selected area electron diffraction (SAED) analyses were performed using TALOSF200S G2 High Resolution Scanning Transmission Electron Microscope (Thermoscientific,USA). Prior to that, the few layer MoS_2_ (FLMS) and BMS powder were mixed in ethanol separately, and a single drop of this solution was dropped on a carbon-coated copper grid. The field effect scanning electron microscopy (FESEM) images of the BMSand FLMS were taken by JEOL-JEM-2100. The Fourier transmission infrared (FTIR) data were taken using an FTIR spectrometer (Perkin Elmer, model: spectrum 2) between 400 cm^-1^ to 4000 cm^-1^. The Raman characterization was measured by using Renishawinvia confocal Raman spectrometer. A smartphone took the digital images of the thin films at different conditions. Then, the red (R), green (G), and blue (B) of those digital images were calculated from Matlab software.

***Gas Sensing Measurement****:*

After depositing two metal electrodes of Ag (*r*=2 nm), the resistive-based gas sensor device was constructed using the thermal evaporation method. The device's two circular electrodes were spaced apart by 4 mm. In order to increase contact quality, the device was heated to 35 °C for 2 minutes in a N_2_ gas atmosphere. In a gas detecting chamber, the gas sensing behavior of a FLMS film-based gas sensor was investigated using various quantities of pure N_2_ gas. The temperature inside the chamber was kept at 35 °C while the ambient temperature remained constant. The same circumstances and room temperature were used for all of the gas sensor measurements. Here, the Keithley 4200 semiconductor characterization equipment was used to detect the variation in the device's current at a bias voltage of 2V.

Figure S1: Resistance vs. time profile at 120 ppm for (a) O_2_, (b) CO_2_, (c) N_2_ gas sensing at RT.

**Theoretical Methodology:**

Density functional theory (DFT) calculations were performed using the Quantum Espresso package^1^. The generalized gradient approximation (GGA) was employed using the Perdew-Burke-Ernzerhof (PBE)^2^ function, which also includes the electronic exchange and correlation energy throughout the work. To account for long-range van der Waals (vdW) dispersion, the Grimme DFT-D3 vdW correction^3^ scheme is also employed. Plane wave pseudopotentials were taken from the publicly available standard solid-state pseudopotentials (SSSP)^4,5^ library. The wave functions were expanded with a plane wave basis set with a kinetic cutoff energy of 48.9 Ry and the Gaussian smearing method was used in all DFT calculations. The Brillouin zone (BZ)integration was conducted using the Monkhorst-Pack^6^ scheme with a 4 × 4 × 1 k-point grid for most of the calculations. All calculations were carried out using a 4 × 4 defected MoS_2_ supercell with a single sulfur vacancy (SV) consisting of 47 atoms (16 Mo and 31 S atoms). To avoid the interaction between two periodic images of different layers, a vacuum spacing of 15 Å is maintained along the non-periodic direction (along the Z axis). The Broyden^5^-Fletcher^8^-Goldfarb^9^-Shanno^10^ (BFGS) algorithm is used to relax the structures with a force tolerance of 0.001 Ry/Bohr. The adsorption energy(*E_ads_*) is calculated using the following scheme:

$$E_{ads}= E_{\mathrm{Mo}S_{2}(SV)+N_{2}}-E_{\mathrm{Mo}S_{2}(SV)}-E_{N_{2}}$$

Where, $E_{\mathrm{Mo}S_{2(SV)}+N_{2}}$, $E_{\mathrm{Mo}S_{2(SV)}}$, and $E_{N_{2}}$denote the total energy of N_2_ adsorbed defectedMoS_2_(SV)monolayer, MoS_2_(SV)monolayer, and N_2_ gas molecule, respectively. A negative value for *E_ads_* indicates that the process is thermodynamically favorable. The charge transfer between the surface and the adsorbed molecules was quantified by means of Bader analysis.^11^

For reference, similar calculations were also performed with a perfect site 4 × 4 MoS_2_ supercell (16 Mo and 32 S atoms).

**Figure S2:** The structure of the perfect MoS_2_ monolayer from (a) side view and (b) top view. The cyan and yellow balls represent Mo and S atoms, respectively, Band structures for the (c) MoS_2_ monolayer and (d) N_2_ adsorbed MoS_2_ monolayer. The cyan line indicates the Fermi energy level.

As shown in Figure S2(a), the MoS_2_ monolayer is a three-atomic layered structure where sheets of Mo atoms are sandwiched between two layers of S atoms. The Mo-S bond distance in the optimized geometry is found to be 2.41 Å, and the angles between S-Mo-S and Mo-S-Mo are found to be 82.47˚ and 82.77˚, respectively, which are consistent with the earlier studies.^12^ The lattice parameter is estimated to be 3.17Å, which is in good agreement with the experimental results (3.16Å).^13^ These results suggest that the pseudopotentials used in the calculations are good enough to study the system.

The band structure of the MoS_2_ monolayer has been shown in Figure S2(c). The direct band gap between the valence band maximum (VBMAX) and the conduction band minimum (CBMIN) is found to be located at the K k-point of the BZ, which is about 1.65 eV. The value is in good agreement with the previous theoretical results (1.64-1.67 eV)^14,15^ and slightly underestimates the experimental findings (1.80 eV).^16^

**Reference**

1. Giannozzi *et al.* QUANTUM ESPRESSO: A Modular and Open-Source Software Project for Quantum Simulations of Materials. *J. Phys.: Condens. Matter.*2009,*21* (39).

10.1088/0953-8984/21/39/395502.

2. Perdew, J. P.; Burke, K.; Ernzerhof, M. *Generalized Gradient Approximation Made Simple*; 1996.

3 .Grimme, S.; Antony, J.; Ehrlich, S.; Krieg, H. A Consistent and Accurate Ab Initio Parametrization of Density Functional Dispersion Correction (DFT-D) for the 94 Elements H-Pu. *J.Chem. Phys.*2010,*132* (15).

https://doi.org/10.1063/1.3382344.

4. Prandini, G.; Marrazzo, A.; Castelli, I. E.; Mounet, N.; Marzari, N. Precision and Efficiency in Solid-State Pseudopotential Calculations. *NPJ Comput. Mater.*2018,*4* (1). https://doi.org/10.1038/s41524-018-0127-2.

5. Lejaeghere*et. al*Reproducibility in Density Functional Theory Calculations of Solids. *Science* 1979,*351* (6280).

https://doi.org/10.1126/science.aad3000.

6. Monkhorst, H. J.; Pack, J. D. *Special Points for Brillonin-Zone Integrations**; 1976; Vol. 13.

7. Broyden, C. G. *The Convergence of a Class of Double-Rank Minimization Algorithms 1. General Considerations*; 1970; Vol. 6. https://academic.oup.com/imamat/article/6/1/76/746016.

8. Fletcher70_BGFS.

9. Goldfarb, D. *A Family of Variable-Metric Methods Derived by Variational Means*.

10. Shanno, D. F. *Conditioning of Quasi-Newton Methods for Function Minimization*; 1970; Vol. 24.

11. Sanville, E.; Kenny, S. D.; Smith, R.; Henkelman, G. Improved Grid-Based Algorithm for Bader Charge Allocation. *J. Comput. Chem.*2007,*28* 899–908.

https://doi.org/10.1002/jcc.20575.

12. Kadantsev, E. S.; Hawrylak, P. Electronic Structure of a Single MoS 2 Monolayer. *Solid State Commun.*2012,*152,* 909–913.

https://doi.org/10.1016/j.ssc.2012.02.005.

13. Yang, D.; Jimenez Sandoval, S.; Divigalpitiya, W. M. R.; Irwin, J. C.; Frindt, R. F. *Structure of Single-Molecular-Layer MoS_2_*; 1991,43.

14. Tang, Q.; Jiang, D. E. Stabilization and Band-Gap Tuning of the 1T-MoS2 Monolayer by Covalent Functionalization. *Chemistry of Materials.*2015,*27* (10) 3743–3748. https://doi.org/10.1021/acs.chemmater.5b00986.

15. Wang, Y.; Li, S.; Yi, J. Electronic and Magnetic Properties of Co Doped MoS2 Monolayer. *Sci Rep.*2016,*6*.

https://doi.org/10.1038/srep24153.

16. Radisavljevic, B.; Radenovic, A.; Brivio, J.; Giacometti, V.; Kis, A. Single-Layer MoS2 Transistors. *Nat Nanotechnology.* 2011,*6* 147–150.

https://doi.org/10.1038/nnano.2010.279.
